# The Complete Chloroplast Genome and Morphological Data Support Reinstatement of *Sedum jinianum* (Crassulaceae) as an Independent Species

**DOI:** 10.1002/ece3.72830

**Published:** 2025-12-26

**Authors:** Lang Shen, Hai‐Jun Ma, Lu‐Jing Wang, Wan‐Zeng Wang, Xiang Wang, Xiang‐Yang Sun, Hao‐Nan Zhang, Wei‐Qi Meng, Kun Liu

**Affiliations:** ^1^ Anhui Provincial Key Laboratory of Biodiversity Conservation and Ecological Security in the Yangtze River Basin, Key Laboratory of Molecular Enzymology and Mechanism of Major Metabolic Diseases College of Life Sciences, Anhui Normal University Wuhu China; ^2^ Institute of Botany, Jiangsu Province and Chinese Academy of Sciences (Nanjing Botanical Garden Mem. Sun Yat‐Sen) Nanjing China; ^3^ Innovative Research Team for Forest Restoration Mechanisms, Chishui National Ecological Quality Comprehensive Monitoring Stations Nanjing Institute of Environmental Sciences, Ministry of Ecology and Environment of the People's Republic of China (MEE) Nanjing China

**Keywords:** Anhui, Crassulaceae, plastome, reinstatement, *sedum*, taxonomy

## Abstract

*Sedum jinianum* (Crassulaceae) has been treated as a synonym of *S. bulbiferum* , a widely distributed species in southern China, in *Flora of China*. However, critical observations on living plants in the wild revealed remarkable morphological distinctions between the two taxa. *Sedum jinianum* can be readily distinguished from *S. bulbiferum* by its alternate leaves, shape and size of leaves, and dark green bulbils. The plastomes of *S*. *jinianum* are 150,041–150,086 bp in length and exhibit a typical quadripartite structure. Phylogenetic analysis based on complete chloroplast genome data indicates that *S*. *jinianum* forms a monophyletic clade, sister to a well‐supported clade comprising four *S. bulbiferum* samples from geographically different populations. These findings support the reinstatement of *S*. *jinianum* as an independent species. Since the original holotype of *S. jinianum* could not be found in the ANUB, a neotype (*L. Shen 240510* at ANUB) is designated here, along with an emended species description.

## Graphical abstract

Phylogenetic analysis based on complete plastome data indicates that S. jinianum forms a monophyletic clade, sister to a well‐supported clade comprising four *S. bulbiferum* samples, supporting the reinstatement of S. jinianum as an independent species.

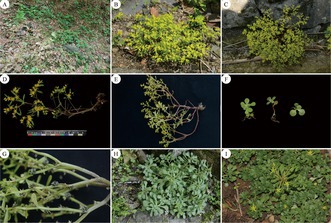

## 1 Introduction

*Sedum jinianum* Guo (1996) was described from Ningguo county in south Anhui province, China, with the designated holotype deposited at ANUB. In the protologue, the author stated that this species resembles *S. bulbiferum* Makino (1891) in that the lower leaves of the stem are short and small with a length of no more than 1 cm, the upper leaves are 1.5–3.5 cm long, linear‐spatulate or linear‐oblong‐lanceolate, the scales are obovate‐trapezoidal rectangles, the sepals are of unequal size, spatulate‐oblong‐lanceolate, 1–5 mm long, and the apex is slightly concave. However, Fu Kunjun (Fu Kun‐tsun) and Hideaki Ohba argued that *S*. *jinianum* lacked the original materials, and the protologue provided insufficient evidence to justify its recognition as a distinct species. Consequently, they treated it as a synonym of *S. bulbiferum* in *Flora of China* (Fu and Ohba 2001).

Our critical observations on living plants in the wild indicated *S*. *jinianum* is morphologically remarkably different from *S. bulbiferum* by an array of characters. We conducted morphological comparisons and molecular phylogenetic analysis to elucidate that *S. jinianum* should be treated as an independent species.

## 2 Materials and Methods

### 2.1 Morphological Comparison

Specimens and individual plants of *S. jinianum* from the wild populations were collected for morphological comparison. Some individuals from the wild were also transplanted and cultivated in the Botanical Garden, Anhui Normal University, Wuhu, for chloroplast genome analysis and further observation. Morphological observation was undertaken using more than 10 living individuals collected in the field or cultivated at the Botanical Garden.

### 2.2 Phylogenetic Reconstruction

Three representative individuals from different populations were selected for further molecular experiments, including one from Jingxian County, Xuancheng City (*H*. *J*. *Ma* 240423), one from Shitai County, Chizhou City (*L*. *Shen* 240510), and the other from Ningguo County, Xuancheng City (*K. Liu* 250401). Three individuals of *S. bulbiferum* from different populations were also included for plastome sequencing. Voucher specimens were deposited in the herbarium of Anhui Normal University (ANUB) (Table 1). Fresh leaves of these individuals were collected and sent to the sequencing company along with ice packs for Next‐generation sequencing (NGS). About 4 GB of raw data for each individual was generated. Chloroplast assembly tests were performed with the most commonly used chloroplast assembly software, Getorganelle (Jin et al. 2020) respectively (Getorganelle tests the K‐mer setup: 21, 25, 39, 45, 51, 65, 85, 105, 121, 127). Using the online tool GeSeq (https://chlorobox.mpimp‐golm.mpg.de/geseq.html) (Tillich et al. 2017) annotated the assembly results (annotated reference genome: *S. bulbiferum* NC_065835). An online website Chloroplot (https://irscope.shinyapps.io/Chloroplot/) (Zheng et al. 2020) was used to draw the circular map of the complete chloroplast genome.

**TABLE 1:** Origins and vouchers of six newly sequenced *Sedum*.

Order	Species	Localities (voucher)	Coordinates	Altitude (m)	Voucher specimen	GenBank
1	S. jinianum	Mifengdong scenic spot, Maolin Town, Jing County, Xuancheng City, Anhui Province	30.423903° N/118.341850° E	355	H. J. Ma 240,423	PQ877685
2	S. jinianum	Qiupu river scenic area, Qidu Town, Shitai County, Chizhou City, Anhui Province	30.214251° N/117.448037° E	280	L. Shen 240,510	PQ877686
3	S. jinianum	Taohuayuan, Fangtang Town, Ningguo City, Xuancheng City, Anhui Province	30.560634° N/118.725672° E	297	K. Liu 250,401	PV656444
4	*S. bulbiferum*	Taohuayuan, Fangtang Town, Ningguo City, Xuancheng City, Anhui Province	30.560633° N/118.725672° E	297	K. Liu 250,402	PV656445
5	*S. bulbiferum*	Qizhangya scenic spot, Nanhe Town, Yingshan County, Huanggang City, Hubei Province	30.553046° N/115.669351° E	272	K. Liu 250,203	PV656446
6	*S. bulbiferum*	Anhui Normal University, Yijiang District, Wuhu City, Anhui Province	31.290727° N/118.388612° E	9	L. Shen 250,403	PV656447

In order to explore the phylogenetic position of *S. jinianum* in *Sedum*, chloroplast genome sequences of 19 accessions representing 15 *Sedum* taxa and three outgroup species ( *Phedimus aizoon* , *Sinocrassula densirosulata*, *Hylotelephium spectabile* ) were downloaded from the Genbank public database at the National Center for Biotechnology Information (NCBI). The sequences were aligned using MAFFT v.7.402 (Katoh and Standley 2014) and then adjusted manually. The phylogenetic tree was constructed using IQ‐Tree v.2.0.3 (Nguyen et al. 2015) by executing 5000 replicates of SH approximate likelihood ratio test (SH‐aLRT) and ultrafast bootstrap (UFBS) (Hoang et al. 2018). Finally, the tree file was visualized by the online tool of Interactive Tree Of Life (iTOL) v5 (Letunic and Bork 2021).

### 2.3 Simple Sequence Repeat (SSR) Analysis

SSRs were identified using the Misa‐web‐IPK Gatersleben online platform (https://webblast.ipk‐gatersleben.de/misa/). We detected mono‐, di‐, tri‐, tetra‐, penta‐, and hexanucleotide repeats among three *S. jinianum* and three *S. bulbiferum* accessions, with minimum repeat thresholds set to 10, 5, 4, 3, 3, and 3 units, respectively.

### 2.4 Non‐Synonymous (K_A_ ) and Synonymous (K_S_ ) Substitution Rate Analysis (K_A_ /K_S_ )

We estimated the relative rates of sequence divergence in both *S. jinianum* and *S. bulbiferum* using KaKs_Calculator v2.0 (Wang et al. 2010) with the chloroplast genome of *Phedimus aizoon* as a reference. The same individual functional protein‐coding exons were extracted for calculating the synonymous (K_S_) and non‐synonymous (K_A_) substitution rates.

### 2.5 Single Nucleotide Polymorphism (SNP) Analysis

Chloroplast genome data of the three *S*. *jinianum* accessions aligned in FASTA format were uploaded to the Galaxy platform (https://usegalaxy.org/), using *S. bulbiferum* as the reference. SNP sites were identified and their genomic positions were extracted using bcftools query.

## 3 Results and Discussion

### 3.1 Morphology

Morphologically, although they both have bulbils, *Sedum jinianum* can be easily distinguished from *S. bulbiferum* by its dark green and incurved bulbils (vs. green and slightly incurved), alternate leaves (vs. proximal stem leaves opposite, distal stem leaves alternate), short pedicels at primary branches and secondary branches (vs. almost sessile) (Table 2). Furthermore, the leaves of *S. jinianum* are usually longer than those of *S. bulbiferum* (1.5–3.0 cm vs. 1.0–1.5 cm), the sepals are usually wider (1–3 mm vs. 1–1.2 mm), and branches with 1 to 4 (vs. long, unbranched stems) (Table 2).

**TABLE 2:** Morphological comparison of *Sedum jinianum* and *S. bulbiferum* .

Characters	*S*. *jinianum*	*S. bulbiferum*
Rosette leaves during florescence	Absent	Absent
Sterile stem	Absent	Absent
Fertile stem	Solitary, purple red and ribbed	Clustered, purple‐red or slightly red and smooth
Leaf blade	Spatulate or obovate	Ovate‐spatulate
Phyllotaxy	Alternate	Proximal stem leaves opposite, distal stem leaves alternate
Leaf size (cm)	(1.5–3.0) × (0.4–0.7)	(1.0–1.5) × (0.2–0.4)
Rhizome	Absent	Short, prostrate
Pedicel	Short pedicels at primary branches and secondary branches	Almost sessile
Anther color	Brownish yellow	Yellow
Sepals	Lanceolate, 5–6 × 1–1.5 mm, spurred	Lanceolate‐oblanceolate, 3–4 × 1–1.2 mm, spurred
Bulbil	Dark green and incurved	Green and slightly incurved
Flowering stage	March–April	April–May
Fruit stage	April–May	May–June

### 3.2 Phylogeny Based on Chloroplast Genome

**FIGURE 1:**
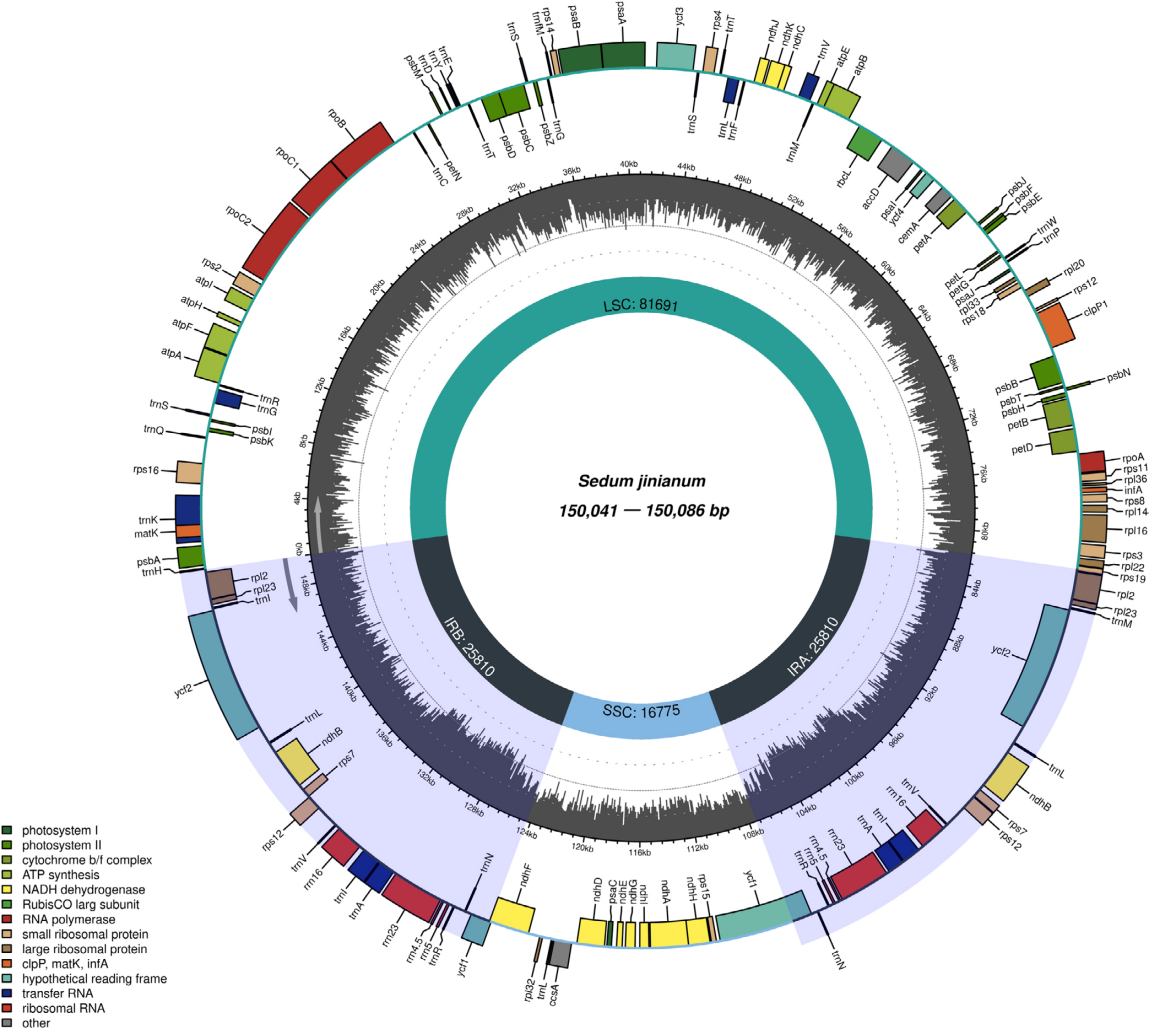
Plastome map of *Sedum jinianum*. Genes belonging to different functional groups are color‐coded. IR, inverted repeat; LSC, large single copy; SSC, small single copy.

**FIGURE 2:**
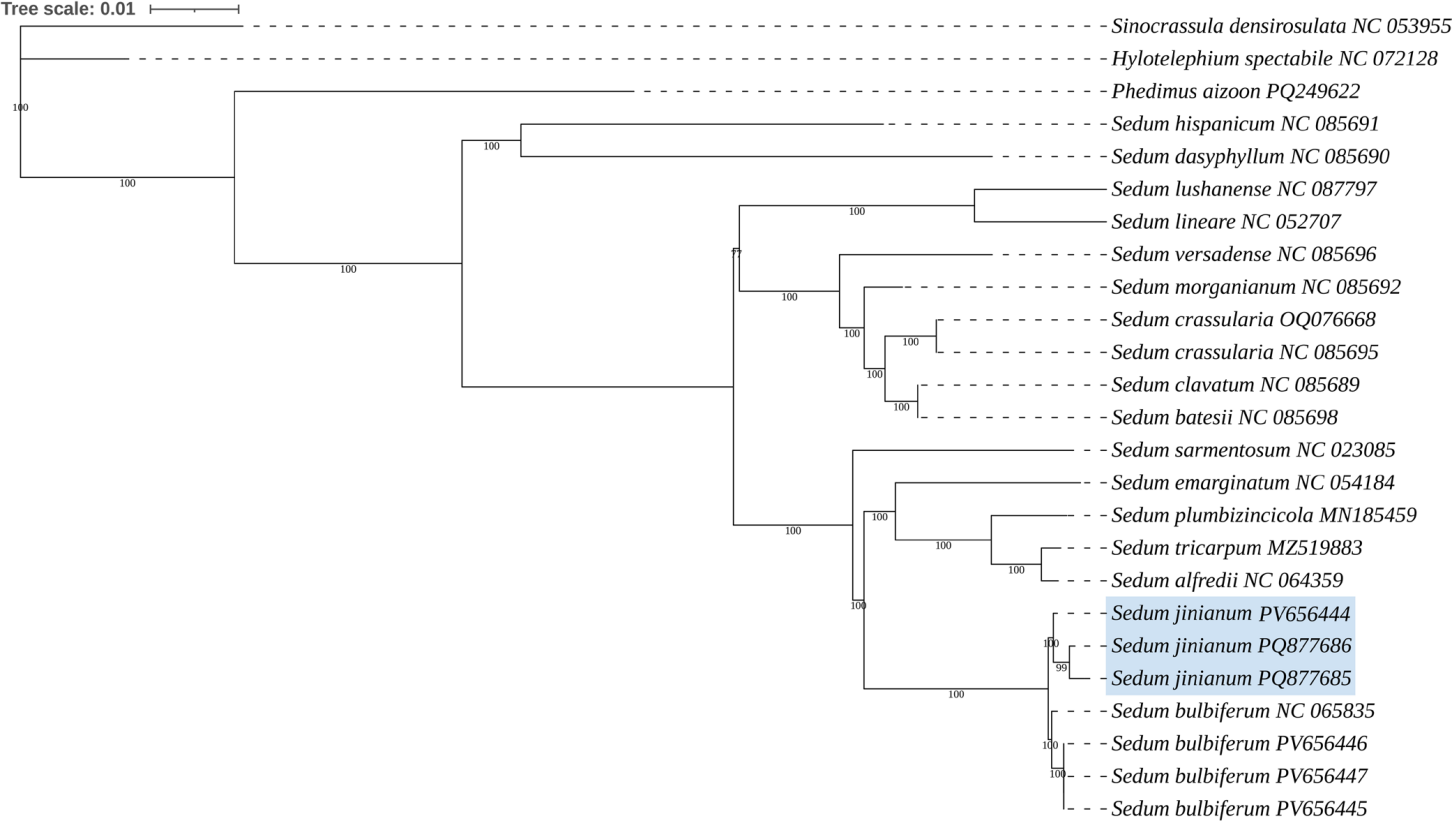
ML phylogenetic tree of 16 *Sedum* species based on the complete chloroplast genome dataset. Numbers beside the nodes are bootstrap values from the ML method. Note that samples of *S. jinianum* are marked in color.

In recent years, phylogenies based on chloroplast genome data have served as key evidence for the species delimitation (Ding et al. 2024; Jiang et al. 2024). The chloroplast genomes of *S. jinianum* were about 150 kb in length, with a typical quadripartite structure consisting of a large single copy region and a small single copy region separated by two long inverted repeats (Figure 1). This structure is congruent with that of other published *Sedum* chloroplast genomes released in GenBank. Phylogenetically, *S. jinianum* is closely related to *S. bulbiferum* (Figure 2). The maximum likelihood (ML) tree inferred from complete plastomes strongly indicates *S*. *jinianum* as a distinct lineage; all three accessions formed a monophyletic clade (MLBS = 100%) as sister to a well‐supported clade comprising four *S. bulbiferum* samples from geographically distant populations (MLBS = 100%) (Figure 2).

### 3.3 SSR Polymorphisms

Using the MISA‐web, we detected 280 SSRs (length ≥ 10 bp) in the plastomes of the three *S. jinianum* and three *S. bulbiferum* accessions, with each accession harboring 46 to 47 SSRs (see Table S1). Mononucleotide repeats composed solely of A or T repeat units were the most abundant, accounting for approximately 83.6% of all SSRs. Notably, all mono‐ and dinucleotide repeats were exclusively composed of A/T units.

### 3.4 Synonymous (K_S_ ) and Non‐Synonymous (K_A_ ) Substitution Rate Analysis

The K_A_ and K_S_ values of the three *S. jinianum* accessions and three *S. bulbiferum* accessions ranged from 0.00122 to 1.08913 and from 0.00248 to 3.67531, respectively. The corresponding K_A_/K_S_ values varied between 0.01153 and 1.07680 (see Table S2). The *rpl2* gene showed the highest values for both K_A_ and K_S_. In contrast, the lowest K_A_ values were observed in *psbD*, while the lowest K_S_ values were found in *ndhB*. Notably, the K_A_/K_S_ ratios of *ndhB* sequences of all six accessions exceeded one, suggesting that positive selection may have driven beneficial variations in the *ndhB* gene of *S. bulbiferum* and *S. jinianum* compared to *P. aizoon* .

### 3.5 SNP Analysis

The results of SNP analysis showed that, compared with *S. bulbiferum* , the three *S. jinianum* accessions contained 106, 109, and 106 SNPs, respectively (see Table S3). The number of transitions (Ts) ranged from 47 to 56, while the number of transversions (Tv) ranged from 53 to 59 across these accessions. The vast majority of SNPs were located in intergenic regions, accounting for 77.4%, 77.1%, and 77.4% in the three *S. jinianum* accessions, respectively. In contrast, only 17.9% to 18.3% of SNPs were found in gene coding regions, and 4.6% to 4.7% were located in tRNA genes.

## 4 Taxonomic Treatment

### 4.1 *Sedum jinianum* X. H. Guo., Acta Bot. Yunnan. 18: 297. 1996. Figures 3, 4, 5

**FIGURE 3:**
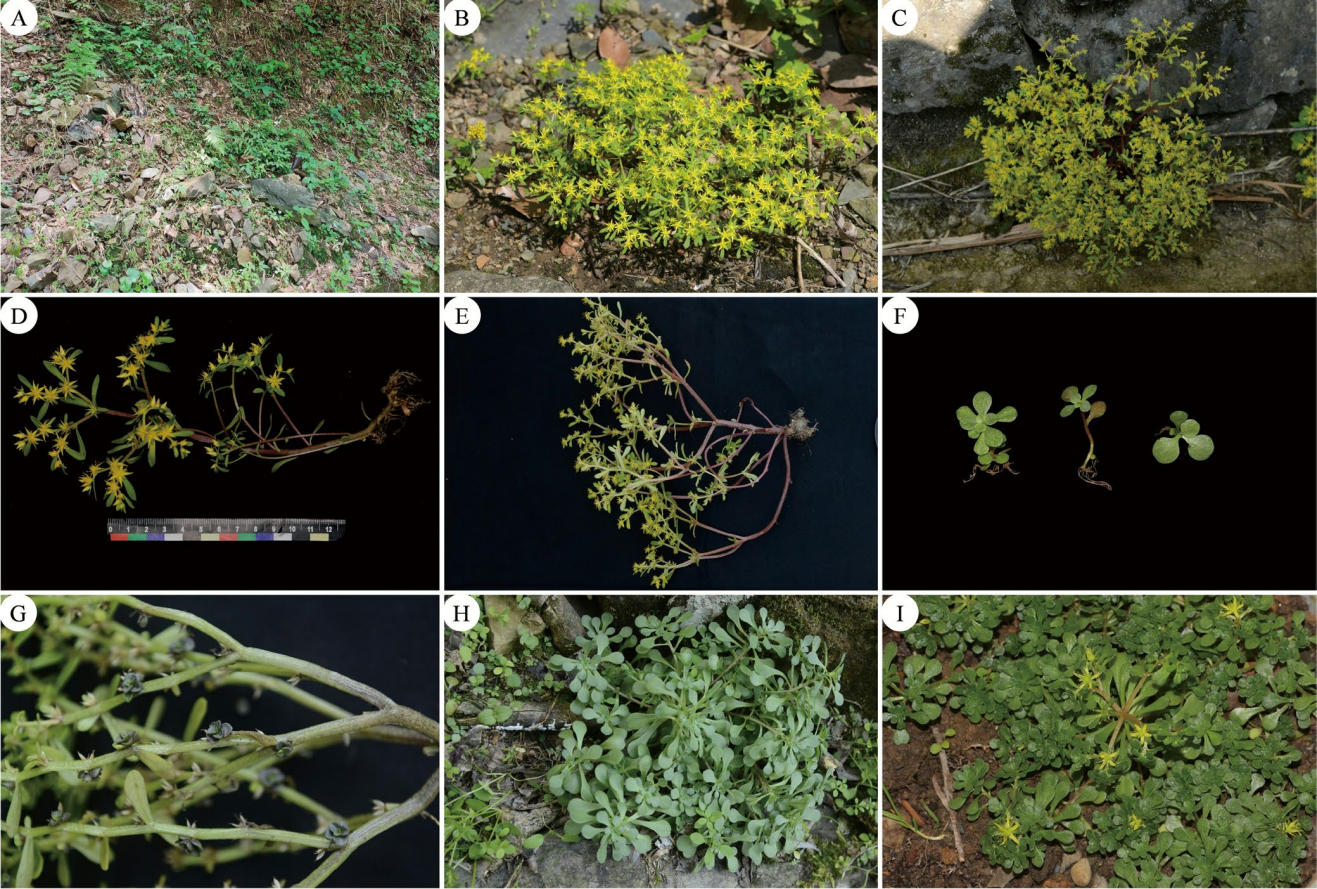
Habit and habitat of *Sedum jinianum* in Jing County. (A) habitat (PQ877685). (B, C) flowering individuals on barren stone habitat. (D, E) flowering individual (PQ877685). (F) juvenile individual. (G) an individual with many bulbils in flower stems. (H) An individual during the vegetative growth stage (PQ877685). (I) part of the flowering individuals in the cultivated environment.

**FIGURE 4:**
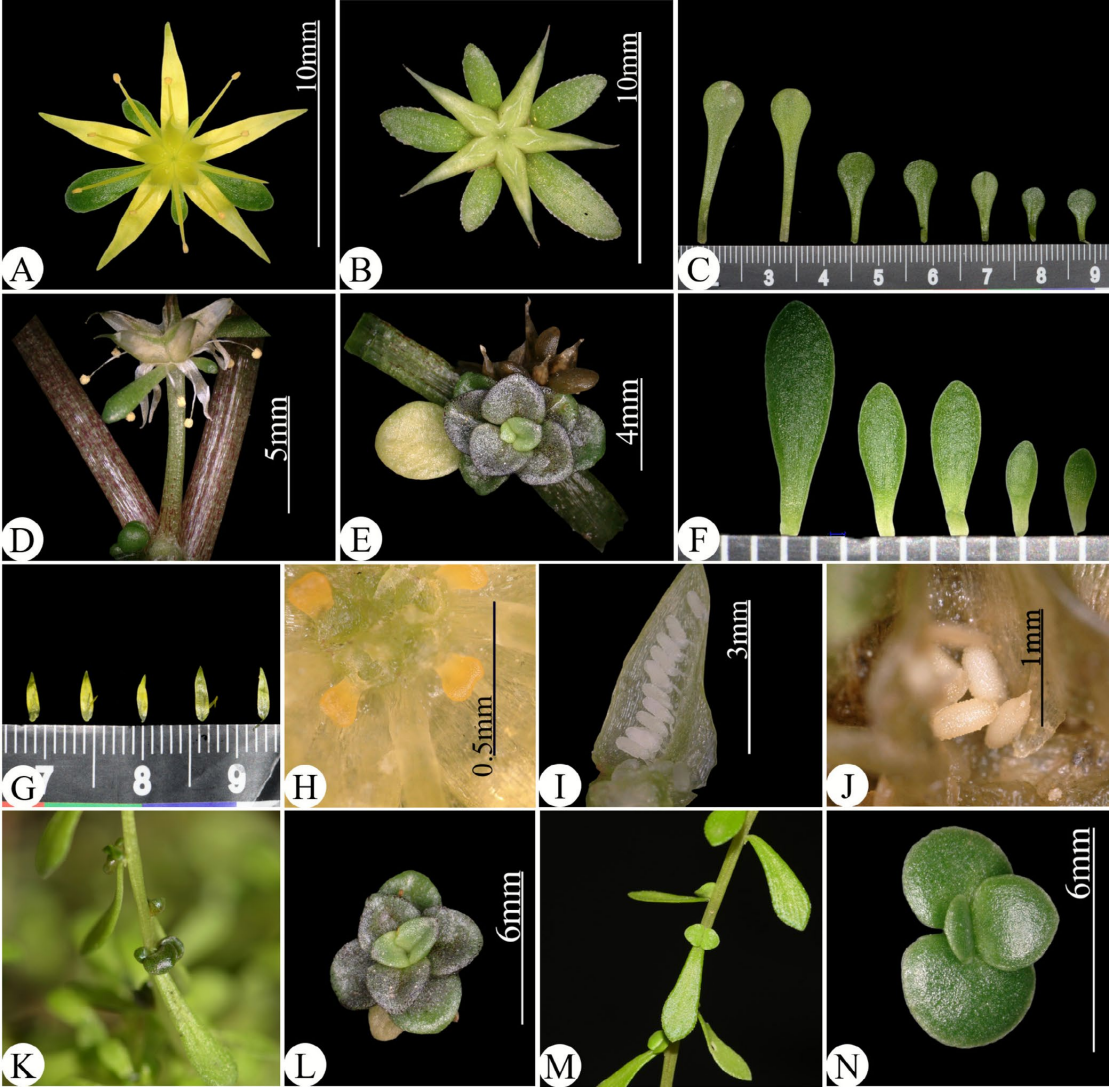
*Sedum jinianum* (PQ877685). (A) Flower. (B) Immature follicle. (C) Leaves. (D) Pedicel. (E) Bulbil. (F) Sepals. (G) Petals. (H) Glands. (I) Immature seeds. (J) Seeds. (K) The side of the bulblet of *S. jinianum*. (L) The front side of the bulblet of *S. jinianum*. (M) The side of the bulblet of *S. bulbiferum* (PV656447). (N) The front side of the bulblets of *S. bulbiferum* (PV656447).

**FIGURE 5:**
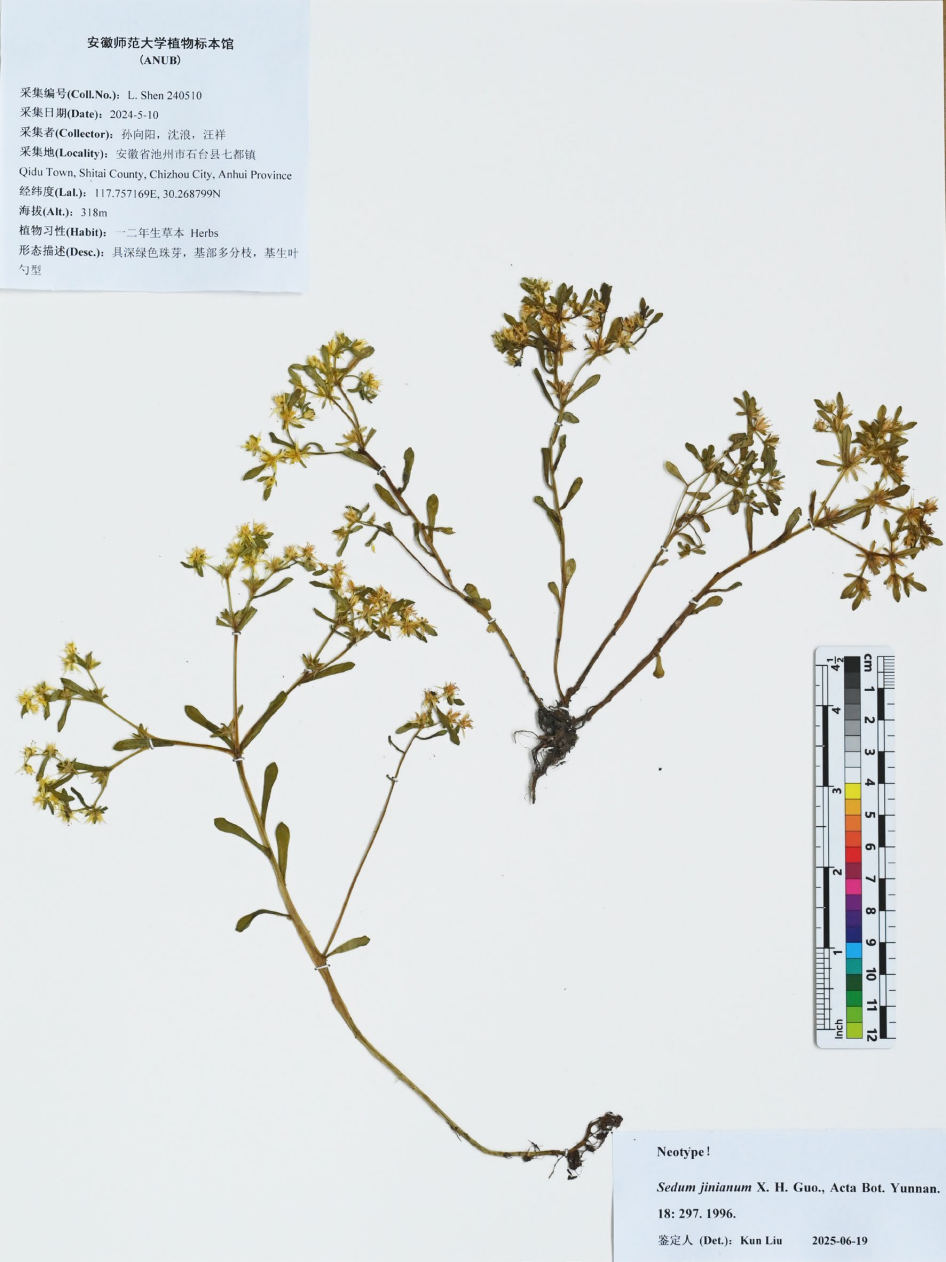
Neotype of *Sedum jinianum* X. H. Guo, *L. Shen 240510* (ANUB).

Type:—CHINA: Anhui: Xuancheng city, Ningguo County, Banqiao, alt. 300–600 m, 16, 5, 1994, Guo Xin‐Hu 94,054. (holotype ANUB (not seen); isotype KUN (not seen)).

### 4.2 Diagnosis

This species can be distinguished from its close relative *S. bulbiferum* by the presence of short pedicels on primary branches, dark green bulbils, alternate spoon‐shaped basal leaves, and lanceolate sepals (Table 2).

### 4.3 Neotype

CHINA. Anhui Province, Chizhou City, Shitai County, Qidu Town, on moist rocks, 117.757169° N, 30.268799° E, 318 m alt., 10 May 2024, L. Shen 240,510 (fl., Neotype ANUB!) (Figure 5).

### 4.4 Description

Biennial herb, glabrous. Roots fibrous. No sterile stems when flowering. Stems solitary, ridged, greenish, often with small purplish dots thus appearing more or less purplish, branched from above the base, 15–30 cm tall, 5–8 mm in diam. Leaves alternate, basal leaves spoon‐shaped, stem‐bearing leaves lanceolate, 15–30 mm long, 4–7 mm wide. Inflorescence in dense terminal cymes, usually 2–3 branched; sepals green, ovate and unequal in length, sepals will become larger after the petals fall off 3–7 mm long, 1–3 mm wide; flowers shortly pedicellate, the pedicels 3–15 mm at primary branches, pedicels of secondary branches 2–12 mm; petals yellow, lanceolate to lanceolate‐oblong, 5–6 × 1–1.5 mm, base connate for 0.1–0.2 mm; stamens 10, yellow, filiform, arranged in 2 whorls, anthers brownish yellow, long ellipsoid; nectar scales orange, spatulate‐quadrangular, 0.3–0.4 × 0.2–0.3 mm, apex obtusely truncate, carpels yellow green, erect, ovoid‐lanceolate, 1.5–2.6 mm long, 0.6–0.9 mm wide, base shortly connate; Seeds numerous, oblong, 0.6–0.8 mm, papillate.

### 4.5 Phenology

Flowering from late March to early April, fruiting from early May.

### 4.6 Distribution and Habitat

**FIGURE 6:**
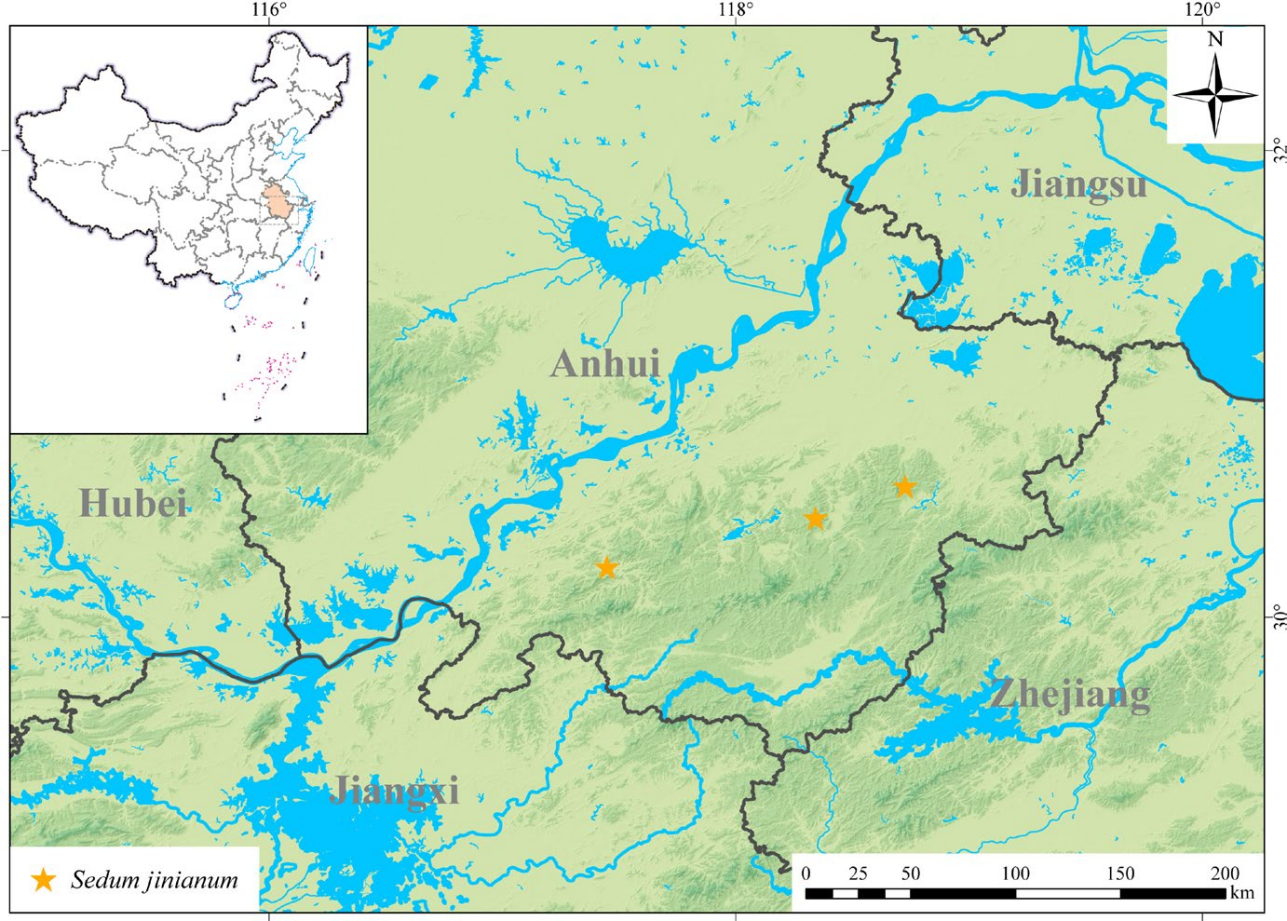
Distribution map of *Sedum jinianum*.

*Sedum jinianum* is widely distributed in southern Anhui (Figure 6). It grows on moist rocks at altitudes of 200–550 m.

### 4.7 Preliminary Conservation Status

*Sedum jinianum* is very common in its type locality and southern regions of Anhui Province. Thus, the conservation status could be considered as Least Concern (LC), according to the IUCN Red List Criteria (IUCN Standards and Petitions Subcommittee 2022).

## Author Contributions

**Lang Shen:** data curation (equal), formal analysis (equal), investigation (equal), software (equal), visualization (equal), writing – original draft (equal). **Hai‐Jun Ma:** data curation (equal), investigation (equal), resources (equal), writing – original draft (equal). **Lu‐Jing Wang:** data curation (equal), investigation (equal), resources (equal). **Wan‐Zeng Wang:** data curation (equal), investigation (equal). **Xiang Wang:** data curation (equal), investigation (equal). **Xiang‐Yang Sun:** data curation (equal), investigation (equal), resources (equal). **Hao‐Nan Zhang:** data curation (equal), methodology (equal), resources (equal). **Wei‐Qi Meng:** conceptualization (equal), methodology (equal), project administration (equal), writing – review and editing (equal). **Kun Liu:** conceptualization (equal), formal analysis (equal), investigation (equal), methodology (equal), project administration (equal), supervision (lead), writing – original draft (equal), writing – review and editing (equal).

## Funding

This study was financially supported by the Provincial Project of Science Research for Colleges and Universities of Anhui Province of China (2023AH050150) and the National Natural Science Foundation of China (31400291).

## Conflicts of Interest

The authors declare no conflicts of interest.

## Supporting information


**Table S1:** Simple sequence repeat (SSR) distribution in the plastomes of three *S. jinianum* accessions and three *S. bulbiferum* accessions.

**Table S2:** The K_A_, K_S_ and K_A_/K_S_ values of three *S. jinianum* accessions and three *S. bulbiferum* accessions.

**Table S3:** Single Nucleotide Polymorphism (SNP) in the plastomes of three *S. jinianum* accessions.

## Data Availability

The plastome sequences generated in this study have been deposited in the National Center for Biotechnology Information (NCBI) database, with GenBank accession numbers provided in Table 1.
